# Supplementary material for: Synergic Effect of Metformin and Everolimus on Mitochondrial Dynamics of Renal Cell Carcinoma
Source: Genes (Basel). 2022 Jul 6;13(7):1211. doi: 10.3390/genes13071211 (PMC9319793; doi:10.3390/genes13071211)
Supplement: Supplementary file 1 [file genes-13-01211-s001.zip › genes-1783462-supplementary.pdf]

Supplementary materials

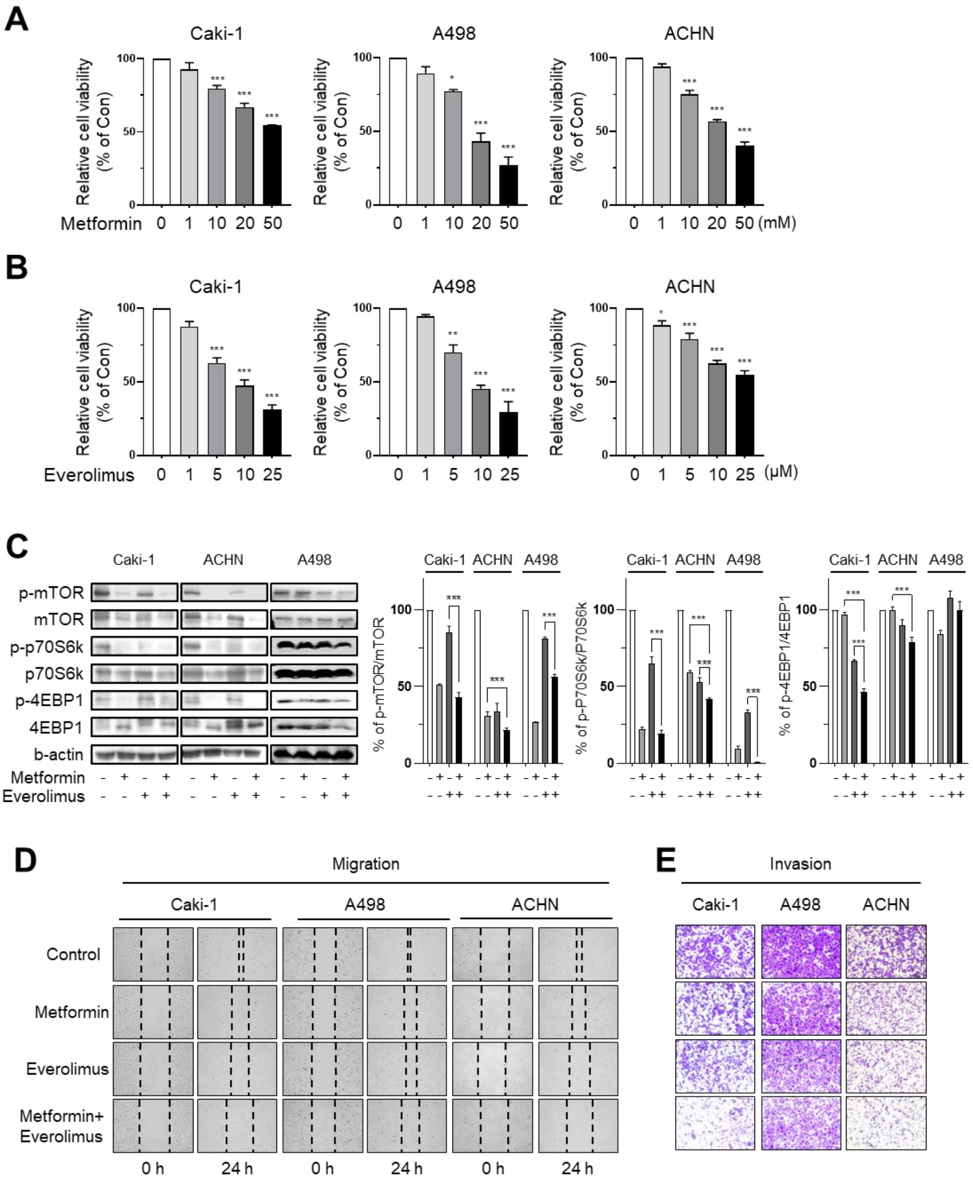

Figure S1. Combination treatment of metformin and everolimus synergistically inhibits RCC. Caki-1, A498, and ACHN cells were incubated (A) in the absence (control) or presence of metformin (1, 10, 20, and 50 mM) for 24 h, and (B) in the absence or presence of everolimus (1, 5, 10, and 25 μM) for 24 h. Cell viability was analyzed using an EZ-cytox™ assay, and the fluorescence values were normalized to the control, and expressed as the percentage of the control. (C) Western blot analysis of the expression of the downstream target proteins of mTOR signaling members (p70S6K and 4EBP1). (D) In vitro scratch migration assay at 0 and 24 h after treatment with the control (absence), metformin (20 mM), and everolimus (10 μM) alone, and a combination of the drugs. (E) Cell invasion assay using Caki-1, A498, and ACHN cells treated with the control (absence), metformin (20 mM), and everolimus (10 μM) separately, and a combination of the drugs. Data are presented as the mean ± standard error of the mean (SEM). *$p < 0.05$; ** $p < 0.01$; and *** $p < 0.001$ vs. control.

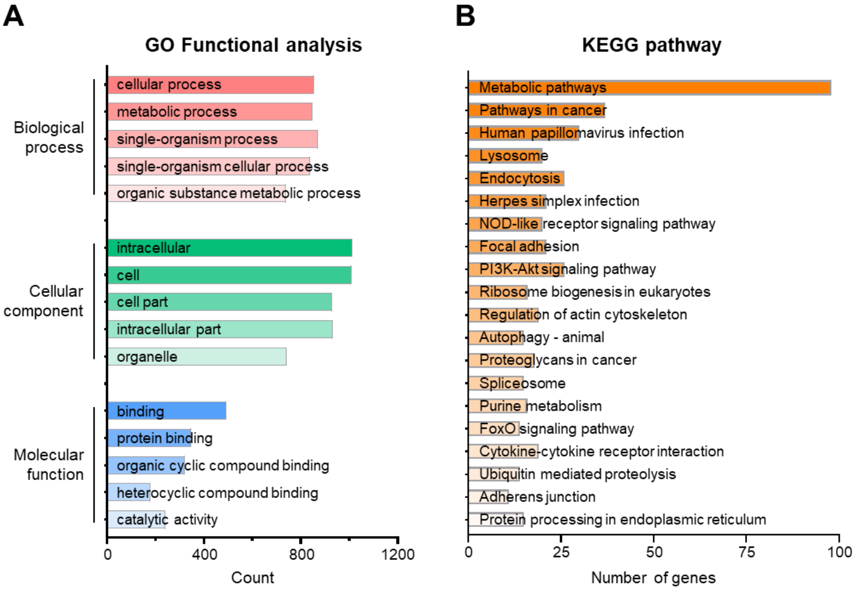

Figure S2. Functional enrichment analysis of six differentially regulated genes related to mitochondrial transporters and mitophagy in RCC: (A) GO analysis showing that six genes were closely corrected with the biological process, cellular component, and molecular function categories; (B) Bar chart showing the enriched pathways from the KEGG analysis. The enrichment in the metabolic pathways, pathways in cancer, and lysosome pathway suggested an optimal correlation with the six differentially regulated genes related to mitochondrial transporters and mitophagy

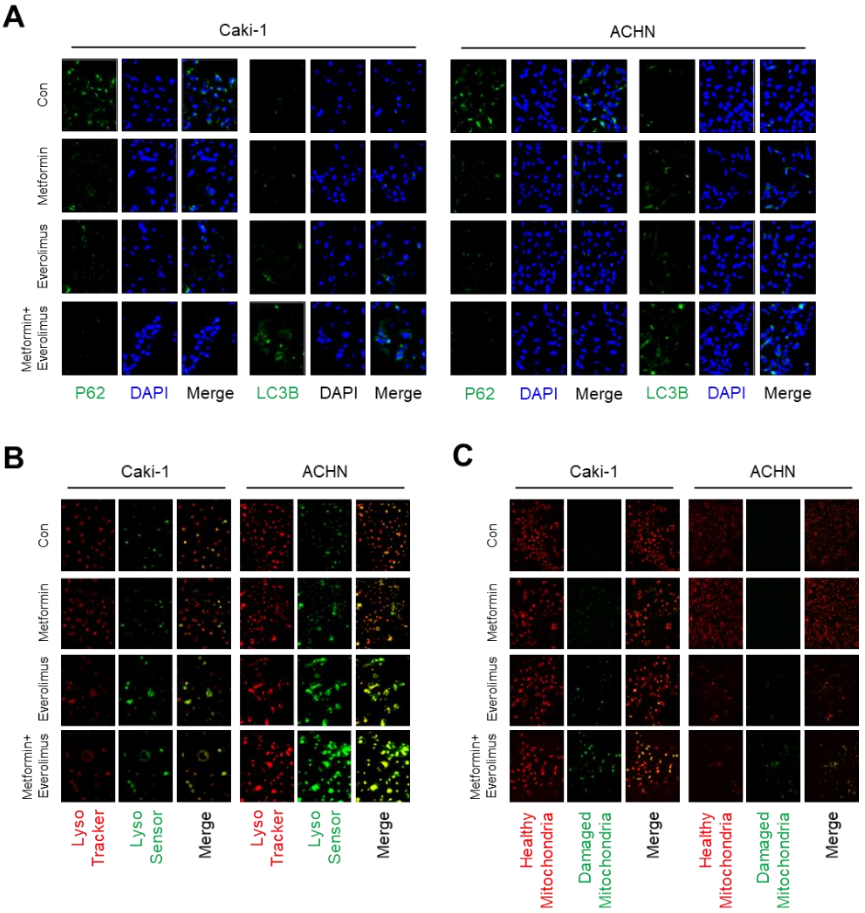

Figure S3. Combination treatment of metformin and everolimus has synergic effects that lead to mitophagy following mitochondrial damage: (A) Representative images of immunocytochemistry analysis with anti-P62 (green), anti-LC3B (green), and DAPI (blue); (B) Cells were incubated with the drug for 24 h, followed by staining with LysoSensor (green) and Lysotracker (red); (C) Fluorescence images of cells stained with JC-1 after treatment according to the drug options (red; healthy mitochondria and green; damaged mitochondria).

Table S1. Sequence of primers used for qRT-PCR.

Primer	Sequence (5′→3′)	Product size (bp)
RT-MFN1-F	GGT GAA TGA GCG GCT TTC CAA G	135
RT-MFN1-R	TCC TCC ACC AAG AAA TGC AGG C	
RT-MFN2-F	ATT GCA GAG GCG GTT CGA CTC A	104
RT-MFN2-R	TTC AGT CGG TCT TGC CGC TCT T	
RT-OPA1-F	GTG GTT GGA GAT CAG AGT GCT G	130
RT-OPA1-R	GAG GAC CTT CAC TCA GAG TCA C	
RT-MIEF2-F	TGT GCT GGG CAT TGC CAC CCT	111
RT-MIEF2-R	TTG AGC AGG CTC AGT TCC TTC C	
RT-DRP1-F	GAT GCC ATA GTT GAA GTG GTG AC	134
RT-DRP1-R	CCA CAA GCA TCA GCA AAG TCT GG	
RT-FIS1-F	CAA GGA ACT GGA GCG GCT CAT T	124
RT-FIS1-R	GGA CAC AGC AAG TCC GAT GAG T	
RT-SLC25A15-F	GGA GAC ATC AGG GAA GAT AGC C	163
RT-SLC25A15-R	GCT CAG TTC ATA GCC ACC GAA G	
RT-SLC25A22-F	GTC AAC GAG GAC ACC TAC TCT G	145
RT-SLC25A22-R	GGA AGT AGA CCA CCT GTG CGA T	
RT-SLC25A30-F	GAT ACC GAG GAA TGT TGC ACG C	106
RT-SLC25A30-R	CCA TAG GAT GCC TGG CGT AAC A	
RT-SLC25A46-F	GGA GTC ACA CTT GGA GCA GAA G	111
RT-SLC25A46-R	GGA TTT CAG TAG AAG GTG TTC TCC	
RT-PINK1-F	GTG GAC CAT CTG GTT CAA CAG G	114
RT-PINK1-R	GCA GCC AAA ATC TGC GAT CAC C	
RT-OPTN-F	ACT CTG ACC AGC AGG CTT ACC T	117
RT-OPTN-R	CTA TGT CAG GCA GAA CCT CTC C	
RT-betaActin-F	CAC CAT TGG CAA TGA GCG GTT C	135
RT-betaActin-R	AGG TCT TTG CGG ATG TCC ACG T	
